# Loss of the spectraplakin gene Short stop induces a DNA damage response in *Drosophila* epithelia

**DOI:** 10.1038/s41598-020-77159-y

**Published:** 2020-11-19

**Authors:** Evan B. Dewey, Amalia S. Parra, Christopher A. Johnston

**Affiliations:** Department of Biology, University of New Mexico, Albuquerque, NM 87131 USA

**Keywords:** Cancer, Cell biology, Developmental biology, Molecular biology

## Abstract

Epithelia are an eminent tissue type and a common driver of tumorigenesis, requiring continual precision in cell division to maintain tissue structure and genome integrity. Mitotic defects often trigger apoptosis, impairing cell viability as a tradeoff for tumor suppression. Identifying conditions that lead to cell death and understanding the mechanisms behind this response are therefore of considerable importance. Here we investigated how epithelia of the *Drosophila* wing disc respond to loss of Short stop (Shot), a cytoskeletal crosslinking spectraplakin protein that we previously found to control mitotic spindle assembly and chromosome dynamics. In contrast to other known spindle-regulating genes, Shot knockdown induces apoptosis in the absence of Jun kinase (JNK) activation, but instead leads to elevated levels of active p38 kinase. Shot loss leads to double-strand break (DSB) DNA damage, and the apoptotic response is exacerbated by concomitant loss of p53. DSB accumulation is increased by suppression of the spindle assembly checkpoint, suggesting this effect results from chromosome damage during error-prone mitoses. Consistent with DSB induction, we found that the DNA damage and stress response genes, Growth arrest and DNA damage (GADD45) and Apoptosis signal-regulating kinase 1 (Ask1), are transcriptionally upregulated as part of the *shot*-induced apoptotic response. Finally, co-depletion of Shot and GADD45 induced significantly higher rates of chromosome segregation errors in cultured cells and suppressed *shot*-induced mitotic arrest. Our results demonstrate that epithelia are capable of mounting molecularly distinct responses to loss of different spindle-associated genes and underscore the importance of proper cytoskeletal organization in tissue homeostasis.

## Introduction

The diverse and complex architectures that form animal tissues require spatial and temporal precision in the cell divisions that underlie their developmental growth patterns and homeostasis throughout adulthood. Due to their ubiquitous nature and association with numerous diseases, epithelia are a particularly important tissue type to understand. Dysregulation in tissue structure, often pursuant to flawed cell divisions, is highly correlated with epithelial-based malignant tumors following transition to a mesenchymal state^1^. Epithelial cell divisions are controlled by a variety of both general and tissue-specific processes. For example, specialized cell adhesion complexes contribute to the cortical polarization along the apical-basal axis. These cortical polarity complexes maintain tissue organization by constraining and orienting cell divisions so as to direct arrangement of daughter cells^2^. As with any cell, general aspects of epithelial cell division are also important to consider. Many of these features, including chromosome segregation, cell cycle checkpoints, and cytokinesis are influenced by the mitotic spindle. The dynamic assembly of this microtubule (MT)-based structure requires dozens of core proteins, and its diverse functions subsequently employ hundreds of additional MT-associated proteins^3^. Defects in bipolar spindle assembly can lead to aberrant chromosome segregation events that induce DNA damage and aneuploidy, both hallmarks of tumor cells^4,5^. Furthermore, the spindle coordinates with the aforementioned cortical polarity cues to direct the orientation of cell division, a critical prerequisite for maintaining tissue structure and suppressing tumor formation^2,6^.

The ability of epithelia to detect and respond to cellular stress is another key aspect of tissue homeostasis. Although cells have evolved mechanisms to resolve certain stressors (e.g. repair pathways that correct DNA damage-induced stress), apoptosis is a dramatic yet common means of eliminating stressed cells. Defects in numerous genes controlling core aspects of epithelia cell function, including cell polarity, spindle assembly and function, as well as oriented cell division have been shown to trigger cell death^7^. In many of these cases, including those involving spindle malfunction, activation of the Jun-related kinase (JNK) appears to be a common signaling effector in the apoptotic response^8^. For example, loss of the centriole duplication factor SAS-4 leads to JNK-dependent cell death in *Drosophila* wing disc epithelia^9^. Spindle assembly is acentrosomal in these cells, leading to DNA damage and spindle misorientation that ultimately triggers JNK-dependent, but p53-indepenent, cell death. Tissue development and architecture are mostly unimpaired, however, due to concomitant JNK-dependent compensatory proliferation^9^. Loss of other spindle-associated genes, including *asterless* and *mud*, as well as cell polarity genes such as *dlg, scrib,* and *par3/*6 also lead to epithelial cell death through similar JNK-mediated mechanisms^7,9–11^. Finally, loss of the spindle assembly checkpoint (SAC) has also been shown to induce JNK-dependent apoptosis in epithelial tissue^12,13^. Interestingly, in some cases JNK functions in a cell non-autonomous manner wherein neighboring cells actively eliminate affected cells to suppress oncogenic growth^14^. Thus, JNK appears to play a central role in epithelial responses to defects in cell polarity and/or division.

In contrast to these results, we recently demonstrated that loss of the spectraplakin gene, Short stop (Shot), induces apoptosis in *Drosophila* wing discs in the absence of JNK activation^15^. Shot is a large actin-microtubule (MT) crosslinking protein that organizes polarized MTs arrays and participates in axon growth, neuromuscular junction maintenance, and cell membrane dynamics^16^. Our study identified several important mitotic functions of Shot, including spindle assembly and orientation as well as chromosome alignment and segregation. Shot localizes to mitotic spindle poles and directly binds Actin-related protein 1 (Arp1), the filamentous component of the Dynein-activating Dynactin complex. In addition to triggering apoptosis in vivo, Shot loss leads to SAC-dependent mitotic delay and DNA segregation errors in cell culture^15^. Many of these mitotic defects are shared with SAS-4 knockdown, yet their apoptotic responses have opposite JNK dependencies. This distinction between otherwise similar phenotypes inspired us to further explore the mechanism for *shot*-mediated apoptosis.

Here we again use the *Drosophila* wing disc as a model epithelial tissue to delineate a molecular mechanism for *shot*-induced apoptosis^17^. We find that Shot knockdown leads to significant DSB accumulation in disc epithelia. Suppression of the SAC increases DNA damage, suggesting it results from defective segregation during mitosis. Interestingly, although Shot loss alone does not activate JNK, concomitant loss of p53 does and also exacerbates the apoptotic response. Shot loss activates an alternative MAP kinase, p38, and we further identify transcriptional activation of GADD45, an important DNA damage response gene in the p53 pathway. Finally, we demonstrate that combined knockdown of Shot and GADD45 in cultured *Drosophila* S2 cells leads to a suppression of mitotic arrest and increased the frequency of DNA segregation errors. Our results demonstrate the ability of epithelia to elicit different responses to loss of genes controlling similar mitotic spindle functions and highlight a JNK-independent mode of epithelial apoptosis following disruption of a cytoskeletal-organizing gene.

## Results

### Shot^RNAi^-mediated apoptosis is selectively associated with p38 activation

**Figure 1:**
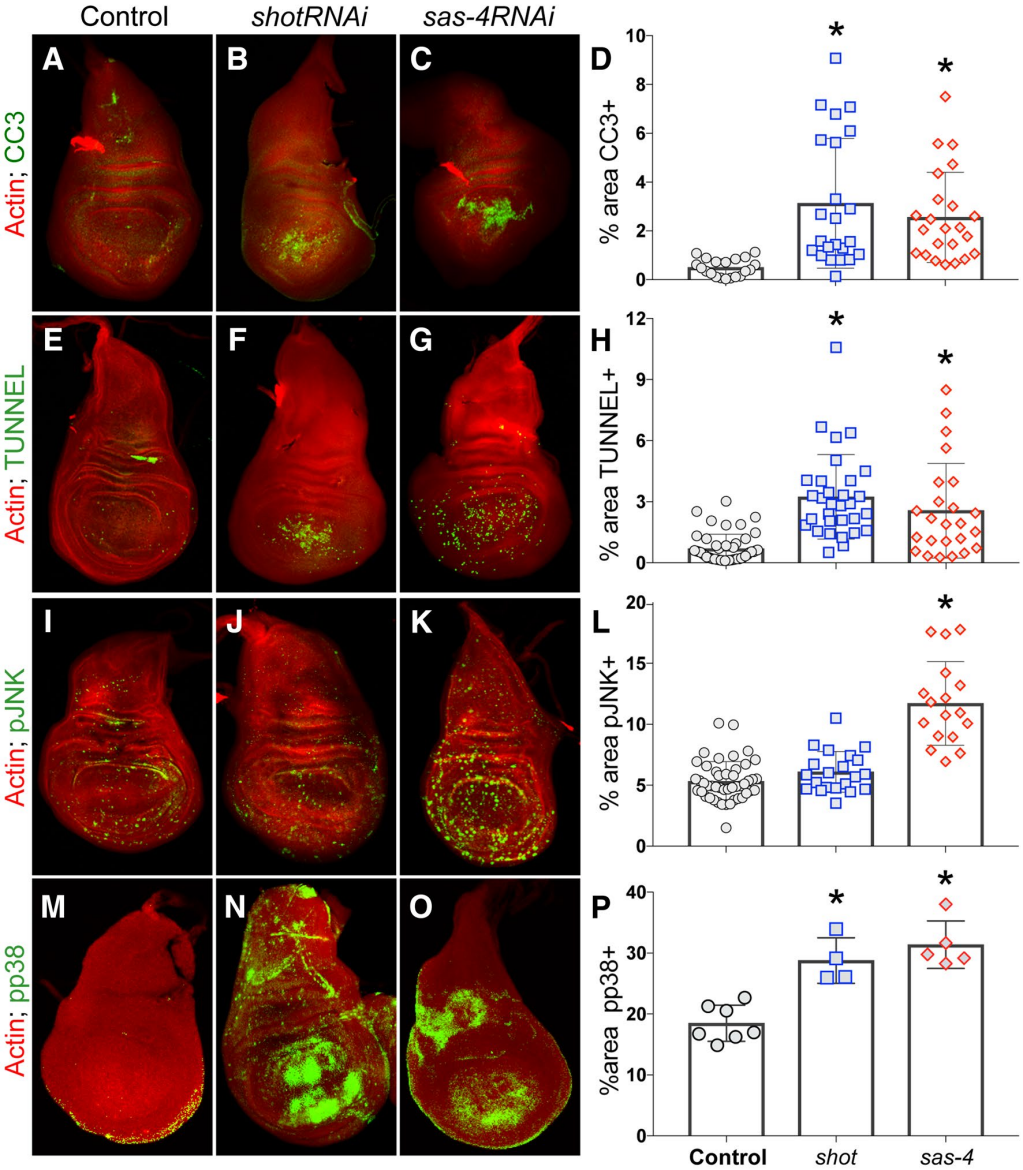
Shot knockdown triggers apoptosis in *Drosophila* wing discs. (**A**–**D**) Control wing discs or those expressing *shot*^*RNAi*^ or *sas-4*^*RNAi*^ were stained with phalloidin (actin; red) and cleaved caspase-3 (CC3; green) to mark apoptotic cells. Images were analyzed for the percent area of the wing pouch positive for CC3. *p < 0.01 compared to Control, ANOVA with Tukey’s post-hoc test. (**E**–**H**) Control wing discs or those expressing *shot*^*RNAi*^ or *sas-4*^*RNAi*^ were stained with phalloidin (actin; red) and TUNNEL (green) to mark apoptotic cells. Images were analyzed for the percent area of the wing pouch positive for CC3. *p < 0.01 compared to Control, ANOVA with Tukey’s post-hoc test. (**I**–**L**) Control wing discs or those expressing *shot*^*RNAi*^ or *sas-4*^*RNAi*^ were stained with phalloidin (actin; red) and active, phosphorylated JNK (pJNK; green). Images were analyzed for the percent area of the wing pouch positive for CC3. *p < 0.01 compared to Control, ANOVA with Tukey’s post-hoc test. (**M**–**P**) Control wing discs or those expressing *shot*^*RNAi*^ or *sas-4*^*RNAi*^ were stained with phalloidin (actin; red) and active, phosphorylated p38 (pp38; green) to mark apoptotic cells. Images were analyzed for the percent area of the wing pouch positive for CC3. *p < 0.01 compared to Control; ANOVA with Tukey’s post-hoc test.

To knockdown Shot expression, we used the *nubbin*-GAL4 wing disc driver to express a short-hairpin interfering RNA (RNAi) directed against the *shot* transcript. This lead to significantly elevated apoptosis, marked by either cleaved-caspase-3 (CC3) or TUNEL staining (Fig. 1A–H). The magnitude of apoptosis was comparable to knockdown of another spindle regulating gene, the centrosomal component *sas-4*. However, whereas *sas-4*^*RNAi*^ lead to an increase in phosphorylated JNK (pJNK), *shot*^*RNAi*^-mediated apoptosis occurred in the absence of such JNK activation (Fig. 1I–L). To investigate this distinction further, we examined the level of activated p38 (pp38), a related MAP kinase that also contributes to apoptosis signaling as well as playing a key role in stress signaling^18^. In this case, *shot*^*RNAi*^ and *sas-4*^*RNAi*^ expression each lead to p38 phosphorylation (Fig. 1M–P). We conclude that distinct mitotic regulators, Shot and SAS-4 specifically, can trigger a common apoptotic response in epithelia but apparently through unique signaling response pathways—while SAS-4 loss activates both JNK and p38 signaling, Shot loss selectively triggers p38 activation.

### Shot^RNAi^ expression leads to a double-strand break DNA damage response

**Figure 2:**
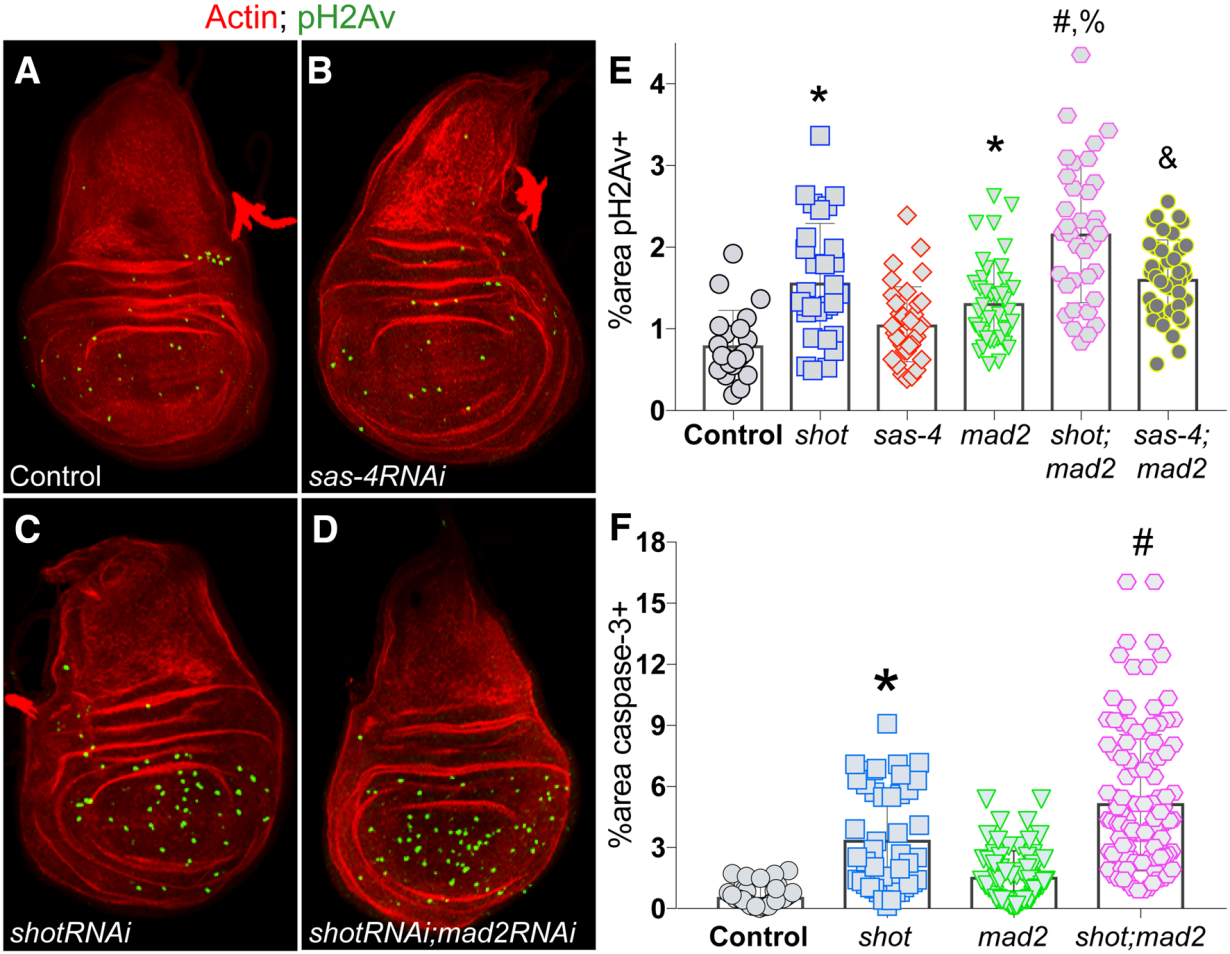
Shot knockdown induces double strand DNA damage in *Drosophila* wing discs. (**A**–**D**) Representative images of wing discs from indicated genotypes stained with phalloidin (actin; red) and phosphorylated H2Av histone (pH2Av; green) to mark cells with DSB-mediated DNA damage. (**E**) Quantification of the percentage of wing pouch area positive for pH2Av signal. *p < 0.01 compared to Control, ^#^p < 0.01 compared to *shot*^*RNAi*^, ^%^p < 0.01 compared to *sas-4*^*RNAi*^*;mad2*^*RNAi*^, ^&^p < 0.01 compared to *sas-4*^*RNAi*^; ANOVA with Tukey’s post-hoc test. (**F**) Quantification of the percentage of wing pouch area positive for cleaved caspase-3 signal. *p < 0.01 compared to Control, ^#^p < 0.01 compared to *shot*^*RNAi*^; ANOVA with Tukey’s post-hoc test.

We next sought to delineate the molecular underpinning for the apoptotic response following *shot*^*RNAi*^ expression. We previously showed that *shot*^*RNAi*^ treatment in cultured S2 cells leads to an elevated incidence of lagging and bridged chromosomes during anaphase^15^. Errors in chromosome segregation are associated with DNA damage and chromosome instability in other cell systems^4,19^, and we thus wondered if *shot* loss might induce these effects in vivo. Using phosphorylated histone γH2Av (pH2Av) as a molecular marker, we found that *shot*^*RNAi*^*-expressing* discs accumulated significant levels of marked DSBs (Fig. 2A,C,E). This effect was not seen in discs expressing RNAi against *sas-4*, however, suggesting DNA damage may be a discriminating factor in the differential apoptotic profiles in the two genotypes (Fig. 2B,E). It should be noted that elevated pH2Av has been reported previously following *sas-4*^*RNAi*^ expression^9^, although this occurred independent of p53 signaling in contrast to *shot*^*RNAi*^ (see below). That study also used an alternative wing disc driver (*apterous*^*GAL4*^) that drives expression in a different region of the tissue than the *nubbin*^*GAL4*^ used here. When *shot*^*RNAi*^ was combined with RNAi against *mad2*, an important component of the spindle assembly checkpoint (SAC), the level of pH2Av was significantly exacerbated (Fig. 2D,E). The percentage of CC3-marked apoptotic cells were also increased in the *shot*^*RNAi*^*;mad2*^*RNAi*^ background (Fig. 2F). These results suggest that DNA damage induced by *shot*^*RNAi*^ is due to errors in chromosome segregation during mitosis and that the SAC normally assists in suppressing these events to prevent cell death.

### p53 loss potentiates Shot-mediated apoptosis and leads to JNK activation

**Figure 3:**
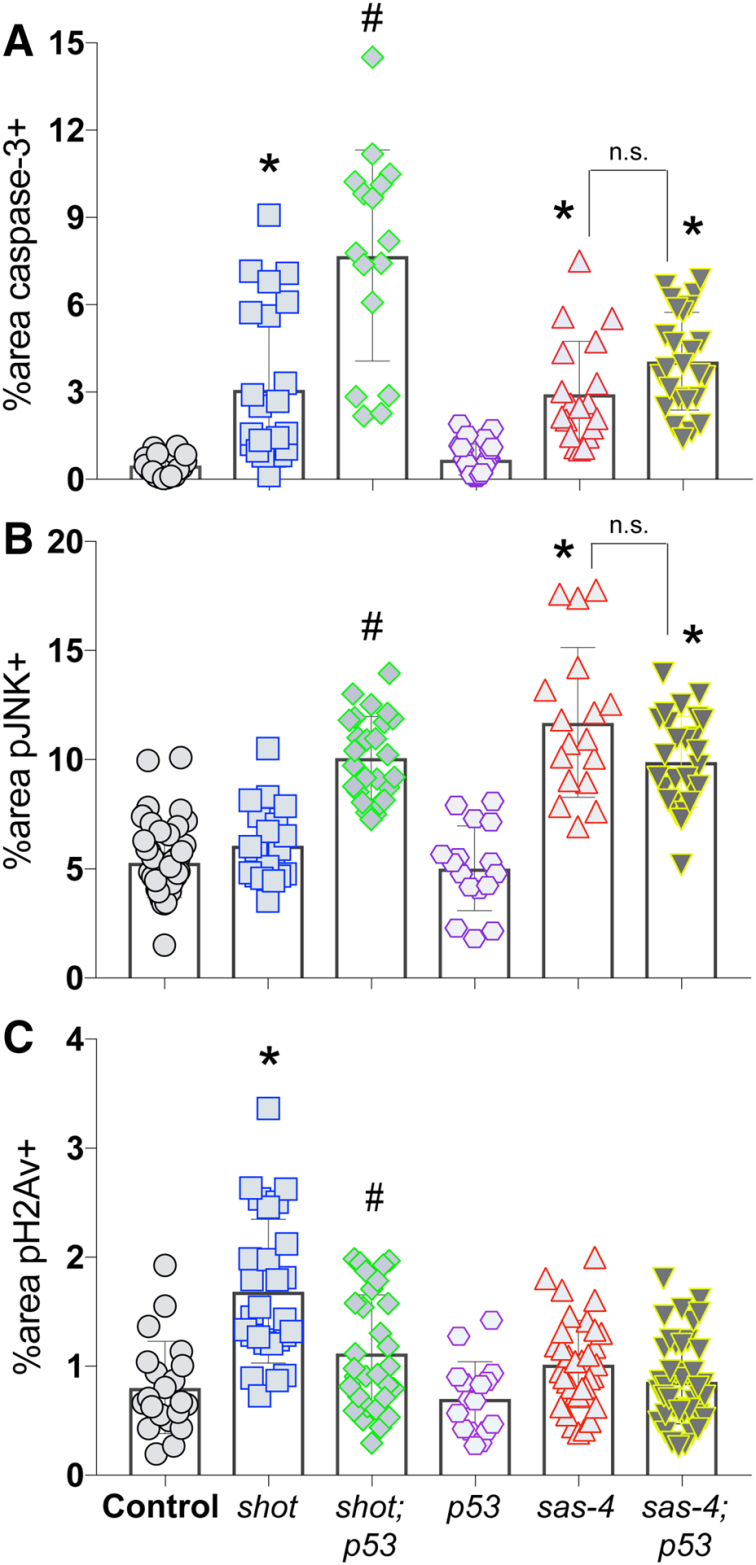
Co-knockdown of p53 and Shot leads to JNK activation and exacerbated apoptosis in *Drosophila* wing discs. (**A**) Quantification of the percentage of wing pouch area positive for cleaved caspase-3 signal. *p < 0.01 compared to Control, ^#^p < 0.01 compared to *shot*^*RNAi*^; ANOVA with Tukey’s post-hoc test. (**B**) Quantification of the percentage of wing pouch area positive for active, phosphorylated JNK signal. *p < 0.01 compared to Control, ^#^p < 0.01 compared to *shot*^*RNAi*^; ANOVA with Tukey’s post-hoc test. (**C**) Quantification of the percentage of wing pouch area positive for phosphorylated H2Av histone. *p < 0.01 compared to Control, ^#^p < 0.01 compared to *shot*^*RNAi*^; ANOVA with Tukey’s post-hoc test.

DNA damage activates a complex signaling response, involving most notably the p53 tumor suppressor protein^20^. To determine the role of p53 in the response to *shot*^*RNAi*^, we generated a *shot*^*RNAi*^*;p53*^*RNAi*^ double transgenic line and compared its responses against a similarly created *sas-4*^*RNAi*^*;p53*^*RNAi*^ line. Unexpectedly, co-depletion of p53 resulted in elevated apoptosis when combined with *shot*^*RNAi*^ (Fig. 3A). This synthetic effect was selective for *shot*, as *sas-4*^*RNAi*^*;p53*^*RNAi*^ discs showing similar CC3 levels as seen in *sas-4*^*RNAi*^ alone tissues. Interestingly, despite the lack of JNK activation in response to *shot*^*RNAi*^ alone, pJNK levels were also significantly elevated in these *shot*^*RNAi*^*;p53*^*RNAi*^ discs to a level similar to *sas-4*^*RNAi*^ discs (Fig. 3B). As with caspase, *p53*^*RNAi*^ did not further increase the *sas-4*^*RNAi*^ pJNK response. Finally, we measured the levels of pH2Av and, again unexpectedly, found that *shot*^*RNAi*^*;p53*^*RNAi*^ discs had reduced levels of marked DSBs relative to *shot*^*RNAi*^ alone (Fig. 3C). Both p53 and JNK signaling pathways are highly intricate^20,21^, making interpretation of these results non-trivial; however, it is likely that they are triggered by an accumulation of genotoxic and oxidative stress in the double mutant discs (see “Discussion” section). We conclude that the classical DNA damage response gene p53 plays an important role in wing disc response to *shot* knockdown by suppressing cell death, likely through error correction.

### Shot^RNAi^ increases GADD45 transcript and alters cell cycle in wing discs

**Figure 4:**
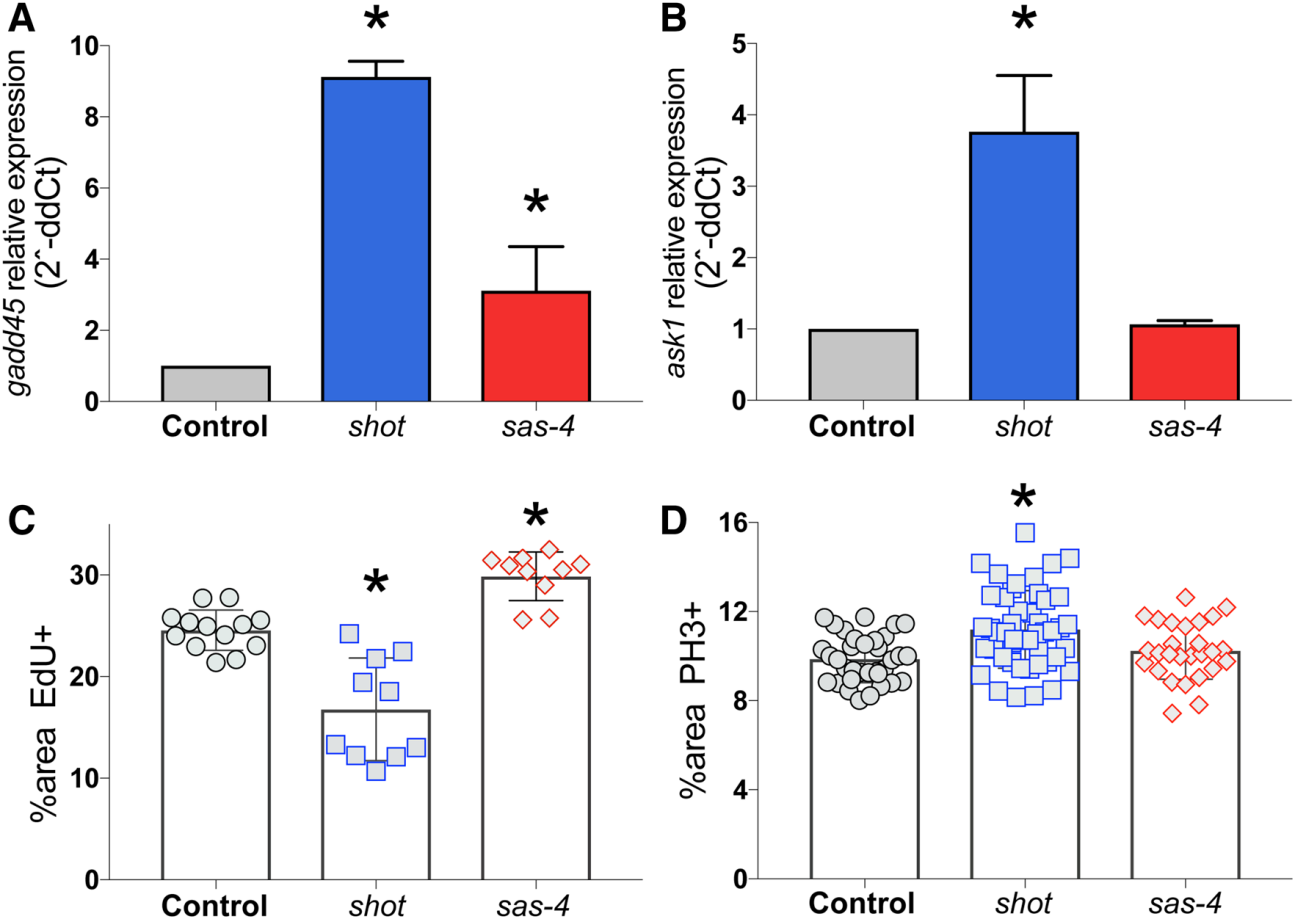
Shot knockdown induces *gadd45* and *ask1* transcriptional upregulation in *Drosophila* wing discs. (**A**) Quantitative PCR targeting *gadd45* using RNA extracted from wing discs of indicated genotypes was conducted and analyzed for fold change relative to Control. Plots show average from 3 independent replicates. *p < 0.05 compared to Control (see “Methods” section). (**B**) Quantitative PCR targeting *ask1* using RNA extracted from wing discs of indicated genotypes was conducted and analyzed for fold change relative to Control. Plots show average from 3 independent replicates. *p < 0.05 compared to Control (see “Methods” section). (**C**) Quantification of the percentage of wing pouch area positive for 5-ethynyl-2′-deoxyuridine (EdU) to mark cell proliferation. *p < 0.01 compared to Control; ANOVA with Tukey’s post-hoc test. (**D**) Quantification of the percentage of wing pouch area positive for phosphohistone-H3 (PH3) to mark mitotic cells. *p < 0.01 compared to Control; ANOVA with Tukey’s post-hoc test.

We next sought to identify additional components involved in the *shot*^*RNAi*^ phenotype, focusing on its distinct DNA damaging effects. GADD45 is a well-established DNA damage response gene that participates in p38 activation and p53-dependent cell cycle arrest and DNA repair^22^. GADD45 is transcriptionally activated in response to growth-arresting DNA damage and cellular stress^23^. To determine if such changes follow Shot knockdown, we extracted wing disc RNA and performed quantitative PCR. As shown in Fig. 4A, *shot*^*RNAi*^-expressing discs showed a significant increase in *gadd45* transcript. Expression of *sas-4*^*RNAi*^ also increased *gadd45*, consistent recent findings^24^, although to a less significant degree than seen with *shot*^*RNAi*^ expression. Furthermore, we found that the level of Ask1 transcript, a DNA damage and stress induced kinase that mediates cell death through p38 signaling^25–27^, was also significantly increased following *shot* knockdown, although it was not significantly changed in discs expressing *sas-4*^*RNAi*^ (Fig. 4B). This result further supports a role of p38 signaling in the wing disc response to Shot knockdown.

We next examined cell proliferation markers in wing discs following *shot* loss to assess potential cell cycle alterations. We found that *shot*^*RNAi*^ expression resulted in a significantly reduced percentage of cells positive for the DNA synthesis marker, EdU (Fig. 4C), indicating reduced proliferation and a lack of compensatory proliferation. Expression of *sas-4*^*RNAi*^, in contrast, increased EdU+ cells, consistent with previous studies showing compensatory proliferation following SAS-4 loss^24^. We next examined the percentage of cells positive for the G2 and M-phase marker phosphohistone-H3 (PH3) and found that *shot*^*RNAi*^ lead to an increase in this case (Fig. 4D). These results are consistent with the known role of GADD45 in G2/M checkpoint induction^28,29^. We found that expression of *sas-4*^*RNAi*^ did not significantly change the percentage of PH3+ cells. Thus, in addition their differential activation of signaling pathways, loss of Shot and SAS-4 also lead to distinct effects on the cell cycle and proliferation dynamics of the wing disc tissue.

### GADD45 knockdown prevents Shot^RNAi^-mediated DSB marks

**Figure 5:**
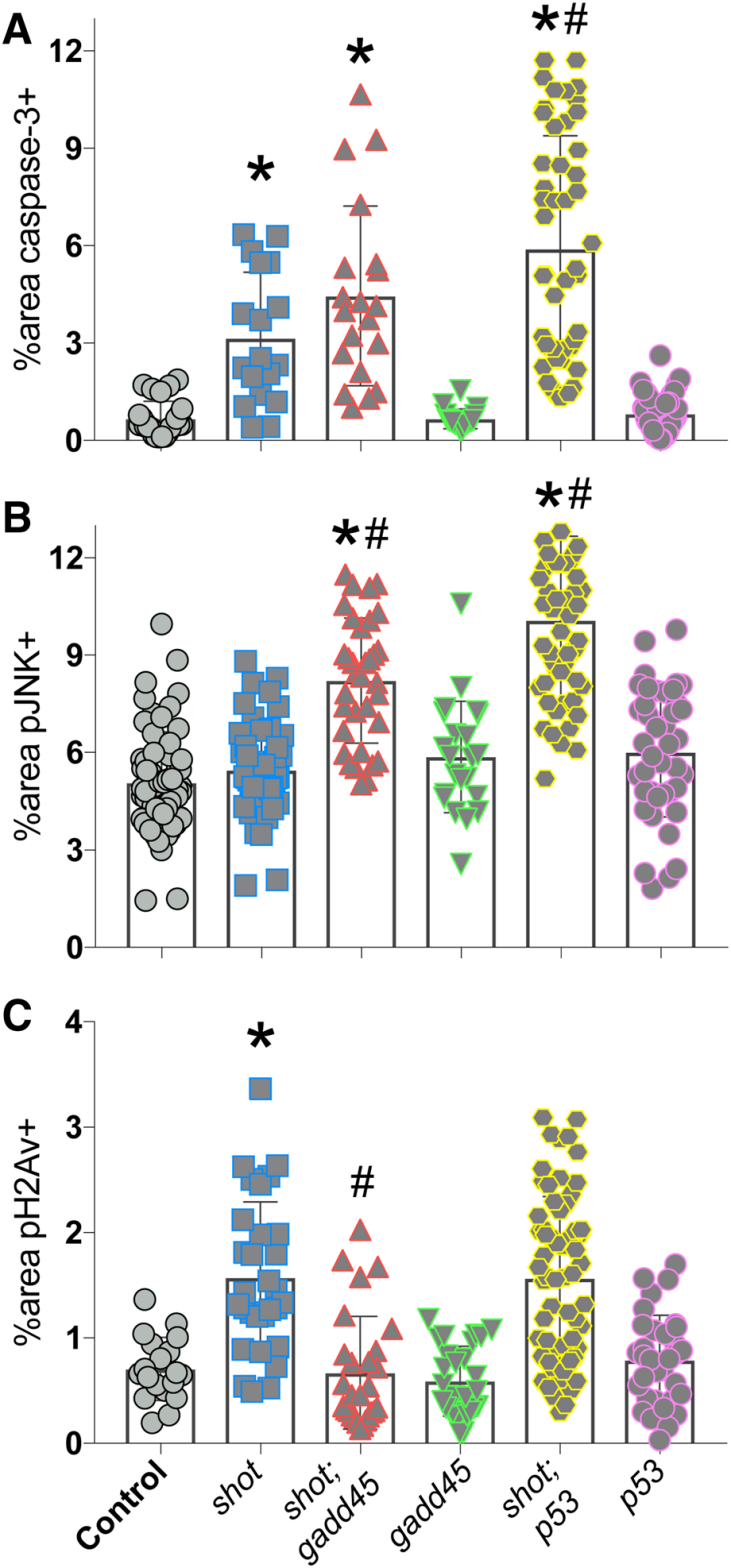
GADD45 contributes to *Drosophila* wing disc response to Shot knockdown. (**A**) Quantification of the percentage of wing pouch area positive for cleaved caspase-3 signal. *p < 0.01 compared to Control, ^#^p < 0.01 compared to *shot*^*RNAi*^; ANOVA with Tukey’s post-hoc test. (**B**) Quantification of the percentage of wing pouch area positive for active, phosphorylated JNK signal. *p < 0.01 compared to Control, ^#^p < 0.01 compared to *shot*^*RNAi*^; ANOVA with Tukey’s post-hoc test. (**C**) Quantification of the percentage of wing pouch area positive for phosphorylated H2Av histone. *p < 0.01 compared to Control, ^#^p < 0.01 compared to *shot*^*RNAi*^; ANOVA with Tukey’s post-hoc test.

To determine what role GADD45 induction plays in the tissue response to Shot knockdown, we generated a *shot*^*RNAi*^*;gadd45*^*RNAi*^ double transgenic line and assessed wing discs for suppression of each specific phenotype described above. These effects were also compared against those in *shot*^*RNAi*^*;p53*^*RNAi*^ discs, as GADD45 and p53 signaling have been linked in several contexts^30^. Somewhat unexpectedly, the level of apoptosis (marked by caspase-3) was not significantly altered in *shot*^*RNAi*^*;gadd45*^*RNAi*^ double knockdown discs compared to *shot*^*RNAi*^ alone, suggesting *shot*^*RNAi*^-induced cell death occurs independent of GADD45 (Fig. 5A). Despite its lack of effect on cell death, the level of activated JNK was increased in *shot*^*RNAi*^*;gadd45*^*RNAi*^ discs compared to Shot knockdown alone. This effect is similar to that seen in *shot*^*RNAi*^*;p53*^*RNAi*^ discs, although p53 knockdown induced a more prominent pJNK response than did GADD45 (Fig. 5B). Although the role of GADD45 in MAPK signaling is complex, our results are consistent with other studies showing it can suppress JNK signaling through inhibition of upstream MKK activity^31–33^. Finally, *shot*^*RNAi*^*;gadd45*^*RNAi*^ discs showed a significant reduction in the level of pH2Av staining, which was indistinguishable from wild-type tissue (Fig. 5C). This result seemingly contradicts the role of GADD45 as a DNA damage response gene, but may instead implicate a role for GADD45 signaling in the maintenance of DSB damage marking. *Gadd45* expression is controlled in part by ATM kinase^34^, but whether GADD45 subsequently facilitates H2A histone phosphorylation by ATM as part of the DNA damage response has not been clarified to our knowledge. GADD45 is also established to promote chromatin accessibility as part of its DNA repair function, the loss of which could also underlie the decreased pH2Av marks in the double mutant discs^35,36^.

Overall, the phenotypes expressed in *shot*^*RNAi*^*;gadd45*^*RNAi*^ discs likely results from a complex signaling network involving interplay among multiple MAPKs, p53, and cell cycle regulatory pathways in response to DNA damage-induced stress^37^. It is also well established that non-autonomous signaling plays an essential role in wing disc responses to cell stress as a mechanism to control tissue growth dynamics^21^, further complicating the interpretation of effects on a tissue-wide scale. Regardless, these results substantiate a role for GADD45 induction in the response to Shot knockdown in discs.

### Shot^RNAi^-mediated cell cycle arrest is GADD45- and Ask1-dependent

**Figure 6:**
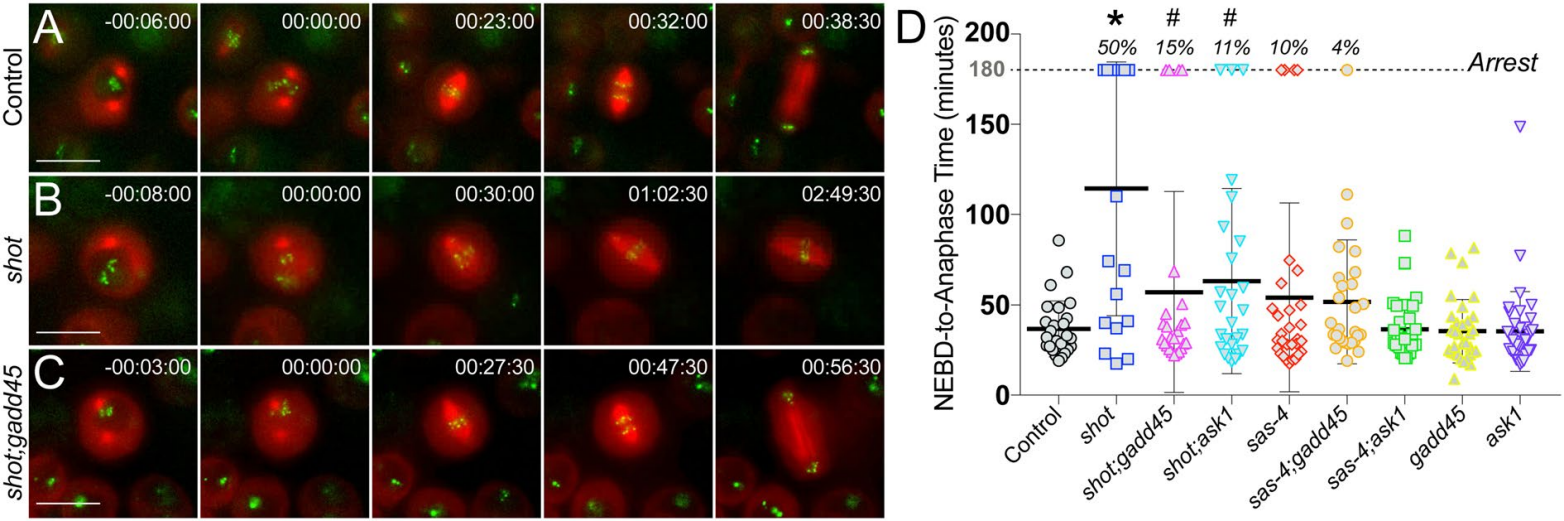
GADD45 and Ask1 are required for cell cycle arrest in *Drosophila* S2 cells following Shot knockdown. (**A**–**C**) *Drosophila* S2 cells stably expressing GFP:CID and mCherry: α-Tubulin were treated without (Control) or with RNAi targeting *shot* and *gadd45*. Cells were imaged from prior to nuclear envelope breakdown (NEBD) through anaphase onset. Images are representative of at least 10 cells. Timestamps are relative to NEBD. (**D**) Plots show timing from NEBD to anaphase onset for each recorded cell for the indicated treatment condition. Cells not progressing into anaphase after 3 h were considered to have undergone mitotic arrest (“Arrest”; indicated by percentages listed at top). *p < 0.01 compared to Control, ^#^p < 0.01 compared to *shot*^*RNAi*^; ANOVA with Tukey’s post-hoc test.

Having assessed the seemingly complex role of GADD45 in tissue-wide responses to Shot knockdown, we next used *Drosophila* S2 cells to determine its effects on individual cell divisions in a simplified culture system. Cell cycle arrest is an important aspect of GADD45 function in both *Drosophila* and human cells^28,38^, thus we began by examining its role in the metaphase arrest previously identified with *shot*^*RNAi*^ treatment in S2 cells^15^. We first compared cell cycle timing (specifically from nuclear envelope breakdown to anaphase onset) following treatment with *shot*^*RNAi*^ or *sas-4*^*RNAi*^ in cells stably expressing mCherry: α-tubulin and GFP:CID (Centromere identifier) to mark the spindle and centromeres, respectively. Compared to Control cells that never arrested, treatment with *shot*^*RNAi*^ induced a significant mitotic delay, with half of cells examined suffering a complete metaphase arrest (Fig. 6A,B,D, Movies 1, 2). Knockdown of *sas-4*, in contrast, caused only a moderate delay that did not reach statistical significance, with fewer cells experiencing full arrest. We then performed co-treatments with RNAi against *shot* and either *gadd45* or *ask1* and found that either combination suppressed the mitotic delay, with a fewer percentage of cells undergoing arrest (Fig. 6C,D, Movie 3). These effects were similar to co-treatment against *mad2*, although in this case cells were never allowed to arrest (not shown, see also^15^). We conclude that *gadd45* and *ask1* are important mediators of cell cycle arrest following knockdown of *shot* in S2 cells. Further, these results help substantiate a model in which these genes may play an important role in the *shot* phenotype in vivo.

### Loss of GADD45 or Ask1 exacerbate Shot-mediated chromosome segregation errors

**Figure 7:**
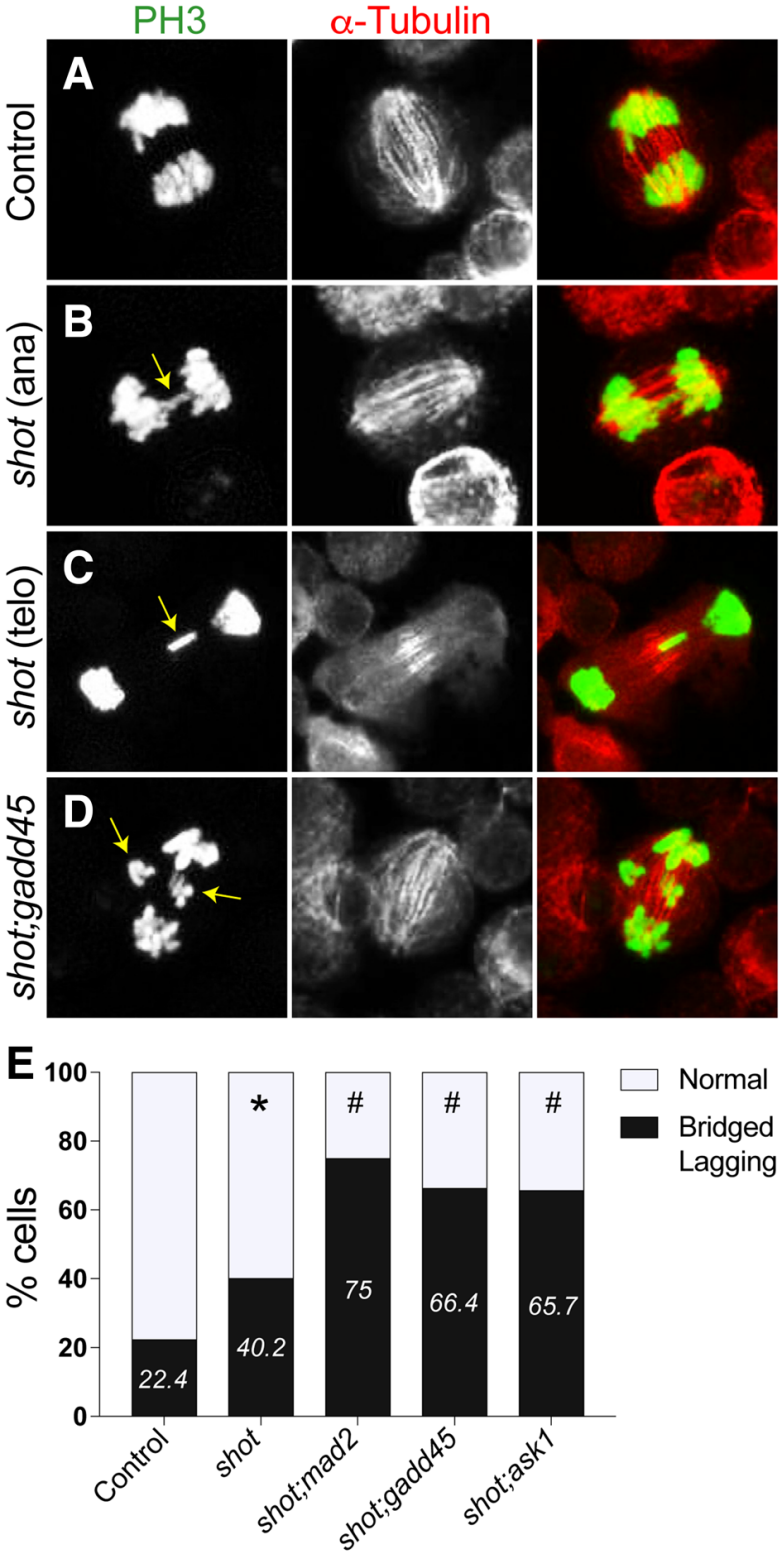
Knockdown of GADD45 exacerbates chromosome segregation defects in *Drosophila* S2 cells with concomitant Shot knockdown. (**A**–**D**) *Drosophila* S2 cells treated without (Control) or with RNAi targeting *shot* and gadd45 were paraformaldehyde fixed and stained for phosphohistone-H3 (PH3, green) and a-Tubulin (red). Yellow arrows indicate lagging or bridged chromosomes in anaphase (ana) and telophase (telo), respectively. Images are representative of at least 30 cells. (**E**) Graph depicts percentage of cells with normal or Bridged/Lagging chromosomes for indicated treatment conditions. *p < 0.01 compared to Control, ^#^p < 0.01 compared to *shot*^*RNAi*^; Fisher’s exact test.

To add further support for the importance of *gadd45* and *ask1*, we next determined how they affect chromosome segregation errors in *shot* depleted cells that escape metaphase arrest. Specifically, we quantified the percentage of cells that experience lagging and/or bridged anaphase chromosomes, erroneous mitotic events associated with DNA pulverization and DSBs^4^. Treatment of S2 cells with *shot*^*RNAi*^ alone led to a near doubling of these events compared to control (40.2% versus 22.4%). Co-treatment targeting *gadd45* or *ask1* significantly increased these events (66.4% and 65.7%, respectively), and against *mad2* showed even further amplification (75%) (Fig. 7). These results suggest that *gadd45* and *ask1* are necessary to limit DNA-damaging segregation errors induced by *shot* loss, likely through checkpoint-mediated mitotic delay and error correction.

## Discussion

Recent studies have shown that direct expression of effector caspases can activate Ask1/p38 signaling through reactive oxygen species accumulation. These signals function autonomously in the apoptotic response as well as non-autonomously in compensatory proliferation to maintain tissue growth^21^. Events upstream of caspases that can trigger such signaling are less clear, however. Here we have compared tissue responses following loss-of-function in two distinct genes, Shot and SAS-4, that are involved in similar functions of the mitotic spindle during cell division. While knockdown of either gene leads to significant apoptosis of wing disc epithelia, these responses are governed by non-identical molecular mechanisms. In the case of *sas-4*, which induces centrosome loss^24^, activation of JNK appears to be the dominant apoptotic signal, a result that agrees with other recent reports^8^, although activation of the p38 MAP kinase was also evident. While loss of Shot also causes p38 activation, it does not lead to JNK phosphorylation (Fig. 1C), suggesting p38 plays a more central role. Shot loss also leads to accumulation of DSB DNA damage, marked by increased phosphorylation of the γH2Av histone variant (Fig. 2). This is consistent with the elevated frequency of chromosome segregation errors and mitotic arrest seen in cultured S2 cells following *shot*^*RNAi*^ treatment (Figs. 6, 7). Interestingly, combined losses of Shot and the DNA-damage response gene p53 lead to elevated cell death and a concomitant increase in JNK phosphorylation. Transcriptional activation of Ask1 and GADD45 genes also appears to play a critical role in the response to Shot loss, likely to induce cell cycle arrest in response to DNA damage. Overall, we demonstrate that wing disc epithelia undergo cell death following loss of key mitotic genes, yet do so through both shared and distinct signaling mechanisms.

Activation of cell death pathways is critical for protecting tissues from the deleterious effects of DNA damage resulting from both intrinsic and extrinsic insults^39^. Erroneous chromosome segregation during cell division, often triggered by defects in mitotic spindle function, has been shown to cause structural abnormalities, DSB accumulation, and genomic instability^4,19^. Our findings are consistent with these and other studies implicating a role of p38, p53, and GADD45 in a complex apoptotic signaling response to DSB-mediated cellular stress (for review see^30^). Interestingly, co-depletion of either p53 or GADD45 together with Shot resulted in several unanticipated findings. Firstly, both of these double knockdown conditions led to elevated apoptosis (although *shot*^*RNAi*^*;gadd45*^*RNAi*^ discs did not reach statistical significance), while reducing the level of pH2Av in the wing disc (Fig. 5). Although seemingly contradictory, it is possible that these effects result from persistent and elevated cellular stress that Shot loss alone was insufficient to induce. The DNA damage response pathway triggers reactive oxygen species production leading to oxidative stress, including in wing discs^40^. Chronic oxidative stress has been shown to increase proteasomal degradation of H2Av, reducing the apparent level of pH2Av marks, and also results in elevated apoptotic cell death^41^. Interestingly, wing discs with a non-phosphorylated H2Av mutant are more sensitive to apoptosis following radiation exposure, further suggesting of an inverse link between H2Av levels and apoptotic cell death^42^. Secondly, both double knockdown conditions also triggered JNK activation not seen in *shot*^*RNAi*^ discs alone (Fig. 5). This is consistent with findings that p53 depletion in wing discs experiencing DNA damage following loss of nucleotide excision repair components also leads unexpectedly to increased cell death that is JNK-dependent^43^. JNK signaling is complex—it contributes to preserving genome stability and can support both proliferative tissue growth and apoptotic cell death^8,44^, thus the exact role of JNK signaling in *shot*-deficient tissue will require further work to clarify. DNA damage following mitotic errors triggers cell cycle arrest and can lead to apoptosis^45^. The suppression of mitotic arrest and increased frequency of chromosomal segregation anomalies following Shot/GADD45 co-knockdown suggests this could lead to elevated stress over time, thereby altering H2Av levels and increasing cytotoxicity.

To conclude, we have revealed a novel role for the cytoskeletal crosslinking agent Shot in maintaining epithelial tissue integrity by preserving genomic stability and suppressing cell death. We previously found that suppressing *shot*^*RNAi*^-induced apoptosis leads to expression epithelial-to-mesenchymal markers, highlight the importance of this cell death response. Here we have molecularly characterized this response, finding that Shot loss leads to DSB damage and activates a seemingly complex signal involving p38 MAP kinase activation, along with p53, GADD45, and Ask1 DNA-damage response genes. Suppression of these effectors prevents cell cycle arrest, exacerbating chromosomal mis-segregation and increasing cell death. Our work complements emerging evidence suggesting a role for cytoskeletal-associated genes in controlling tissue growth dynamics. Future studies will be required to identify other related genes that might function similarly to Shot and to compare the signaling response tissues mount in the event of their dysfunction. It will also be important to determine the potential tumorigenic potential of tissues deficient in Shot function.

## Methods

### Fly stocks

The following stocks were obtained from the Bloomington Stock Center: VALIUM TRiP lines for *shot*^*RNAi*^ (stock # 28336), *sas-4*^*RNAi*^ (stock # 35049), *p53*^*RNAi*^ (stock # 103001), *mad2*^*RNAi*^ (stock # 106003), *gadd45*^*RNAi*^ (stock # 35023), and *nubbin*^*GAL4*^ (stock # 25754). All double transgenic lines were generated using a *Cyo/Br;TM2/TM6* double balancer line (generous gift from Dr. Richard M. Cripps, UNM).

### Imaginal wing disc immunostaining

Immunostaining of imaginal wing discs was performed using methods we have previously described^15^. Briefly, discs were dissected from wandering third instar larvae in PBS. Discs were fixed in 4% paraformaldehyde at room temperature for 20 min with rocking. Following fixation, discs were quickly washed three times in wash buffer (PBS supplemented with 0.3% Triton X-100) and then once at room temperature for 20 min with rocking. Discs were blocked in block buffer (wash buffer supplemented with 1% BSA) for 1 h at room temperature. Phalloidin-568 (1:50, Thermo Fisher) and primary antibodies in block buffer were incubated with constant rocking at 4 °C for 24–48 h. Subsequently, discs were washed and treated with secondary antibodies in block buffer for 2 h at room temperature. Washed discs were mounted in Vectashield Mounting Medium for Fluorescence or 80% glycerol and stored at 4 °C until imaged. Imaging was performed on a Zeiss LSM780 confocal microscope. Antibodies used were as follows: rabbit phosphohistone-H3 (1:1000), rabbit cleaved caspase-3 (1:500, Cell Signaling Technology), mouse MMP-1 (1:100, Developmental Studies Hybridoma Bank), rabbit phospho-JNK (1:1000, Promega), and rabbit phospho-p38 MAKP (1:50, Cell Signaling Technology). All secondaries, preabsorbed and non-crossreactive, were purchased from JacksonImmuno and used at 1:250 dilutions.

Area quantification of wing disc maximum intensity projections was done using thresholding and the ‘thresholdcolour’ plugin in ImageJ. The area of the wing pouch was first taken using the polygon selection tool and recorded in pixels. The extraneous portions of the disc (the hinge and the notum that lie outside of the Nubbin expression pattern) were then removed using the ‘Clear Outside’ command. Then using the ‘Threshold Colour’ command, only pixels displaying green were selected. Using the ‘Threshold’ command, pixels positive for signal were selected and background was excluded. The number of positive pixels were then calculated using the ‘Analyze Particles’ command setting the minimum detectable pixel size to 2 square pixels, and displaying results. The number of pixels obtained was then normalized to a percent area measurement by dividing it by the size of the wing pouch obtained earlier.

### Imaginal wing disc EdU staining

Discs were dissected in Schnieder’s S2 media and then placed in 1 mL of 100 µg/mL 5-ethynyl-2′-deoxyuridine (EdU) in Schnieder’s S2 media for 1 h at room temperature (RT) with nutation. Following EdU labeling, discs were fixed, washed, and blocked as described previously for imaginal disc immunostaining. After the 30 min room temperature (RT) blocking with nutation step, block buffer was removed as completely as possible with a pipette. Then, 500 µL of Click-iT Reaction Cocktail (prepared as described by the Click-iT EdU Imaging Kit Protocol, Invitrogen) was added and discs were incubated for 30 min at RT with nutation, protected from light. Click-iT Reaction cocktail was then removed and discs were washed once quickly with 1 mL block buffer. Primary and secondary antibody staining and disc mounting were then performed as described previously for imaginal disc immunostaining.

### Imaginal wing disc TUNEL staining

Imaginal Discs were stained with Terminal deoxynucleotidyl transferase dUTP nick end labeling (TUNEL) using the Apoptag Red In situ Apoptosis Detection Kit (EMD Millipore) protocol as an additional way to mark apoptotic cells. Discs were dissected as described for other immunostaining procedures through fixation and washing. Following the 20 min room temperature (RT) wash with nutation, wash buffer was removed as completely as possible with a pipette. Discs were then incubated with 150 µL of Equilibration Buffer for 1 h at RT. Equilibration buffer was then completely removed from discs, and 50 µL of Working Strength TdT Enzyme Solution (35 µL of Reaction Buffer and 15 µL of TdT enzyme) was added. Discs were then incubated at 37 °C for 1 h to promote TdT enzyme activity and dUTP labeling. Following this, Working Strength TdT Enzyme Solution was removed as completely as possible, and discs were then quickly washed once with Working Strength Stop/Wash Buffer (1 mL Stop/Wash Buffer in 34 mL dH_2_O) and then again for 10 min with nutation at RT. Working Strength Stop/Wash Buffer was then removed and discs were washed three times quickly with 1 mL Wash buffer and once for 20 min with nutation. Disc blocking and primary antibody staining were then carried out as described for imaginal disc immunostaining until addition of the secondary antibody. Working Strength Rhodamine Antibody Solution (53% [v/v] Blocking Solution and 47% [v/v] Anti-Digoxigenin Conjugate) was added and discs were incubated for 1–2 h at RT with nutation. Additional secondary antibodies were added to the Working Strength Rhodamine Antibody Solution at the dilutions described if needed. After secondary antibody incubation and mounting, the imaginal disc immunostaining protocol was followed as described.

### Real-time quantitative PCR

RNA was extracted from imaginal wing discs using the RNeasy Mini Kit according to manufacturer protocol (Qiagen). Complementary DNA (cDNA) was synthesized using reverse transcriptase and 500 ng RNA in 20 μL reactions according to manufacturer’s instructions with an iScript cDNA Synthesis Kit (Bio-Rad, #170-8891, Hercules, California, U.S.A.). The primers for the candidate genes were designed for a single 100–350 bp region with the Integrated DNA Technologies OligoAnalyzer tool (www.idtdna.com). Primers used were as follows: *shot:* Forward—AGCCAGAGGTCAAGAAACAC, Reverse—GTCAGTTCCACAGCTTGTCT; *sas-4:* Forward—GAGCAAGAGCTGGTCAAGAT, Reverse—CCTCCGATTCCACAGAACTAC; *ask1:* Forward—CTCCACTGAACGACGACAAA, Reverse—CACGATCCCAAATAGCGAACTA; *gadd45:* Forward—AACTGGACCTGGAGCTAGA, Reverse—GACTTGGAGAGCACGTTGAT. Amplification efficiency for each primer pair was established with 1:10 serial dilutions of cDNA. Real-time PCR was performed in 20 μL reactions containing SsoAdvanced Universal SYBR Green Supermix (Bio-Rad Laboratories, #1725271). Real-time PCR was performed using a C1000 Touch Thermal Cycler (Bio-Rad Laboratories) with CFX96 Touch Real-Time PCR Detection System (Bio-Rad Laboratories, #184-5097).

Technical duplicates of each biological triplicate for each treatment were tested. Fold changes between control and RNAi treated groups relative to the reference gene, *D. Melanogaster* Mnf, were calculated using normalized expression (ΔΔ (Ct)) using CFX Manager Software v3.1 (Bio-Rad Laboratories). Unpaired Student’s t-test was used to assess statistical significance of normalized gene expression values, and a p < 0.05 was considered statistically significant.

### S2 cell maintenance and RNAi treatments

*Drosophila* Schneider S2 cells (Invitrogen) were cultured and RNAi-treated using protocols we have previously described^15^. Briefly, S2 cells were grown in Schneider’s insect media (Sigma) supplemented with 10% heat-inactivated fetal bovine serum (SIM). Cells were passaged every 3–4 days and stocks were maintained at 25 °C in the absence of CO_2_.

Primers used for RNAi construction were designed using the SnapDragon web-based service (https://www.flyrnai.org/snapdragon), and all primer synthesis was carried out by Invitrogen. Primer sets that amplify segments of ~ 200–600 base pairs within the coding or 3′-UTR sequence of desired targets were optimized for efficiency and specificity and synthesized with T7 promoter sequence recognition tags. Targeted sequences were designed to universally recognize all possible isoforms for desired transcript. PCR-amplified target sequences were transcribed to yield double-stranded RNA using the Megascript T7 kit (Ambion) following the recommended protocol.

For RNAi treatment, S2 cells were seeded in six-well dishes at 1 × 10^6^ cells per well in 1 mL of serum-free Schneider growth media and incubated with 10 μg of desired RNAi. After 1 h, 2 mL of SIM was added, and cells were incubated for an additional 5 days prior to subsequent assays. Cells were typically supplemented with an additional 0.5–1 mL of SIM following day 3 to avoid excessive evaporation.

### S2 cell immunostaining and live-cell imaging

Both fixed and live-cell imaging of S2 cells were performed using protocols we have previously described^15^. Briefly, S2 cells were treated with or without indicated RNAi and subsequently mixed with fresh SIM in 24-well dishes containing 12 mm diameter round glass coverslips. Cells were incubated for 2–3 h to allow for adherence to poly-l-lysine-treated coverslips and to increase the percentage of mitotic cells. Cells were then fixed using a treatment of 4% paraformaldehyde for 10 min. Fixed cells were washed three times (5 min each) with wash buffer (0.1% Triton X-100 in PBS), followed by a 1-h incubation with block buffer (0.1% Triton X-100 and 1% BSA in PBS). Primary antibodies diluted in block buffer were then incubated with slides overnight at 4 °C. Following primary antibody incubation, slides were washed three times with block buffer. Secondary antibodies were then added and incubated at room temperature for 2 h. Antibodies were removed and slides were washed four times with wash buffer. Finally, coverslips were inverted and mounted using EverBrite Hardset reagent (VWR) and stored at 4 °C prior to imaging.

Primary antibodies used were rat anti-α-tubulin (1:500; Sigma Aldrich) and rabbit anti-PH3 (1:1000; Abcam). Secondary antibodies (preabsorbed and non-crossreactive) were purchased from JacksonImmuno and used at 1:250 dilution. Imaging was performed using Olympus IX83 inverted fluorescence microscopes and collected under oil immersion at × 60 magnification.

Live-cell imaging and movie rendering was done according to our previously published protocol^46^. Briefly, movies were acquired using S2 cells stably expressing an inducible GFP:CID (a generous gift from Dr. Gary Karpen, UC Berkeley), which we subsequently stably transfected with inducible mCherry-α-tubulin (selected for using puromycin resistance) to generate a stable double transgenic S2 cell line. Cells were treated with Control or RNAi as described in the previous section. Upon completion of RNAi treatment, cells were settled at a density of 2 million/mL into Nunc Lab-Tek II 4-chambered coverglass chambers pre-coated with poly-l-lysine. After settling for 1 h, chambers were placed onto an Olympus IX-83 inverted epifluorescent microscope and appropriate cells were located and imaged at either 30 s or 1 min intervals using a Hammatsu Orca-Flash 4.0LT camera, with three z-stacks taken at each interval. If cells (e.g. *shot*^*RNAi*^ treated) did not enter anaphase after 3 h the experiment was stopped and recorded as 180 min data point and considered as a metaphase arrest event. This was done due to significant photobleaching and the potential for phototoxicity. Movies were converted to AVI or MOV files and analyzed using ImageJ.

## Supplementary information

Supplementary Information.

Supplementary Video 1.

Supplementary Video 2.

Supplementary Video 3.

## References

[1] Francou A, Anderson KV (2020). The epithelial-to-mesenchymal transition in development and cancer. Annu. Rev. Cancer Biol..

[2] Ragkousi K, Gibson MC (2014). Cell division and the maintenance of epithelial order. J. Cell Biol..

[3] Petry S (2016). Mechanisms of mitotic spindle assembly. Annu. Rev. Biochem..

[4] Crasta K (2012). DNA breaks and chromosome pulverization from errors in mitosis. Nature.

[5] Ganem NJ, Pellman D (2012). Linking abnormal mitosis to the acquisition of DNA damage. J. Cell Biol..

[6] Lu MS, Johnston CA (2013). Molecular pathways regulating mitotic spindle orientation in animal cells. Development.

[7] McCaffrey LM, Macara IG (2011). Epithelial organization, cell polarity and tumorigenesis. Trends Cell Biol..

[8] Dhanasekaran DN, Reddy EP (2017). JNK-signaling: a multiplexing hub in programmed cell death. Genes Cancer.

[9] Poulton JS, Cuningham JC, Peifer M (2014). Acentrosomal Drosophila epithelial cells exhibit abnormal cell division, leading to cell death and compensatory proliferation. Dev. Cell.

[10] Brumby AM, Richardson HE (2003). Scribble mutants cooperate with oncogenic Ras or Notch to cause neoplastic overgrowth in Drosophila. EMBO J..

[11] Nakajima Y, Meyer EJ, Kroesen A, McKinney SA, Gibson MC (2013). Epithelial junctions maintain tissue architecture by directing planar spindle orientation. Nature.

[12] Clemente-Ruiz M, Muzzopappa M, Milan M (2014). Tumor suppressor roles of CENP-E and Nsl1 in Drosophila epithelial tissues. Cell Cycle.

[13] da Silva SM, Moutinho-Santos T, Sunkel CE (2013). A tumor suppressor role of the Bub3 spindle checkpoint protein after apoptosis inhibition. J. Cell Biol..

[14] Ohsawa S (2011). Elimination of oncogenic neighbors by JNK-mediated engulfment in Drosophila. Dev. Cell.

[15] Dewey EB, Johnston CA (2017). Diverse mitotic functions of the cytoskeletal cross-linking protein Shortstop suggest a role in Dynein/Dynactin activity. Mol. Biol. Cell.

[16] Voelzmann A (2017). Drosophila Short stop as a paradigm for the role and regulation of spectraplakins. Semin. Cell Dev. Biol..

[17] Beira JV, Paro R (2016). The legacy of Drosophila imaginal discs. Chromosoma.

[18] Wagner EF, Nebreda AR (2009). Signal integration by JNK and p38 MAPK pathways in cancer development. Nat. Rev. Cancer.

[19] Janssen A, van der Burg M, Szuhai K, Kops GJ, Medema RH (2011). Chromosome segregation errors as a cause of DNA damage and structural chromosome aberrations. Science.

[20] Kastenhuber ER, Lowe SW (2017). Putting p53 in context. Cell.

[21] La Marca JE, Richardson HE (2020). Two-faced: roles of JNK signalling during tumourigenesis in the drosophila model. Front. Cell Dev. Biol..

[22] Smith ML (1994). Interaction of the p53-regulated protein Gadd45 with proliferating cell nuclear antigen. Science.

[23] Fornace AJ, Alamo I, Hollander MC (1988). DNA damage-inducible transcripts in mammalian cells. Proc. Natl. Acad. Sci. U.S.A..

[24] Poulton JS, McKay DJ, Peifer M (2019). Centrosome loss triggers a transcriptional program to counter apoptosis-induced oxidative stress. Genetics.

[25] Matsuzawa A, Ichijo H (2008). Redox control of cell fate by MAP kinase: physiological roles of ASK1-MAP kinase pathway in stress signaling. Biochim. Biophys. Acta.

[26] Ichijo H (1997). Induction of apoptosis by ASK1, a mammalian MAPKKK that activates SAPK/JNK and p38 signaling pathways. Science.

[27] Chen Z (1999). ASK1 mediates apoptotic cell death induced by genotoxic stress. Oncogene.

[28] Wang XW (1999). GADD45 induction of a G2/M cell cycle checkpoint. Proc. Natl. Acad. Sci. U.S.A..

[29] Zhan Q (1999). Association with Cdc2 and inhibition of Cdc2/cyclin B1 kinase activity by the p53-regulated protein Gadd45. Oncogene.

[30] Salvador JM, Brown-Clay JD, Fornace AJ (2013). Gadd45 in stress signaling, cell cycle control, and apoptosis. Adv. Exp. Med. Biol..

[31] Gupta M, Gupta SK, Hoffman B, Liebermann DA (2006). Gadd45a and Gadd45b protect hematopoietic cells from UV-induced apoptosis via distinct signaling pathways, including p38 activation and JNK inhibition. J. Biol. Chem..

[32] Papa S (2004). Gadd45 beta mediates the NF-kappa B suppression of JNK signalling by targeting MKK7/JNKK2. Nat. Cell Biol..

[33] Ueda T, Kohama Y, Kuge A, Kido E, Sakurai H (2017). GADD45 family proteins suppress JNK signaling by targeting MKK7. Arch. Biochem. Biophys..

[34] Jang ER (2010). ATM modulates transcription in response to histone deacetylase inhibition as part of its DNA damage response. Exp. Mol. Med..

[35] Smith ML (2000). p53-mediated DNA repair responses to UV radiation: studies of mouse cells lacking p53, p21, and/or gadd45 genes. Mol. Cell Biol..

[36] Carrier F (1999). Gadd45, a p53-responsive stress protein, modifies DNA accessibility on damaged chromatin. Mol. Cell Biol..

[37] Liebermann DA, Hoffman B (2008). Gadd45 in stress signaling. J. Mol. Signal.

[38] Camilleri-Robles C, Serras F, Corominas M (2019). Role of D-GADD45 in JNK-dependent apoptosis and regeneration in Drosophila. Genes (Basel).

[39] Borges HL, Linden R, Wang JY (2008). DNA damage-induced cell death: lessons from the central nervous system. Cell Res..

[40] Clemente-Ruiz M (2016). Gene dosage imbalance contributes to chromosomal instability-induced tumorigenesis. Dev. Cell.

[41] Gruosso T (2016). Chronic oxidative stress promotes H2AX protein degradation and enhances chemosensitivity in breast cancer patients. EMBO Mol. Med..

[42] Madigan JP, Chotkowski HL, Glaser RL (2002). DNA double-strand break-induced phosphorylation of Drosophila histone variant H2Av helps prevent radiation-induced apoptosis. Nucleic Acids Res..

[43] Villicana C, Cruz G, Zurita M (2013). The genetic depletion or the triptolide inhibition of TFIIH in p53-deficient cells induces a JNK-dependent cell death in Drosophila. J. Cell Sci..

[44] Girnius N, Edwards YJ, Garlick DS, Davis RJ (2018). The cJUN NH2-terminal kinase (JNK) signaling pathway promotes genome stability and prevents tumor initiation. eLife.

[45] Hayashi MT, Karlseder J (2013). DNA damage associated with mitosis and cytokinesis failure. Oncogene.

[46] Dewey EB, Parra AS, Johnston CA (2019). Use of Drosophila S2 cells for live imaging of cell division. J. Vis. Exp..

