# Supplementary material for: Loss of the spectraplakin gene Short stop induces a DNA damage response in Drosophila epithelia
Source: Sci Rep. 2020 Nov 19;10:20165. doi: 10.1038/s41598-020-77159-y (PMC7677407; doi:10.1038/s41598-020-77159-y)
Supplement: Supplementary file 1 — Supplementary Information. [file 41598_2020_77159_MOESM1_ESM.pdf]

Loss of the spectraplakin gene Short stop induces a DNA damage response in *Drosophila* epithelia

Evan B. Dewey, Amalia S. Parra, and Christopher A. Johnston

SUPPLEMENTARY INFORMATION

Movie 1 – Control S2 cell division

S2 cells stably expressing GFP:CID and mCherry:α-Tubulin were treated in the absence of RNAi and subsequently imaged from just prior to NEB through telophase.

Movie 2 – *shot*^{RNAi}-treated S2 cell division

S2 cells stably expressing GFP:CID and mCherry:α-Tubulin were treated with *shot*^{RNAi} and subsequently imaged from just prior to NEB through telophase.

Movie 3 – *shot*^{RNAi}/*gadd45*^{RNAi}-treated S2 cell division

S2 cells stably expressing GFP:CID and mCherry:α-Tubulin were treated with a combination of *shot*^{RNAi} and *gadd45*^{RNAi} and subsequently imaged from just prior to NEB through telophase.
